# Stability properties of PrP^Sc^ from cattle with experimental transmissible spongiform encephalopathies: use of a rapid whole homogenate, protease-free assay

**DOI:** 10.1186/1746-6148-9-167

**Published:** 2013-08-15

**Authors:** Catherine E Vrentas, Justin J Greenlee, Thierry Baron, Maria Caramelli, Stefanie Czub, Eric M Nicholson

**Affiliations:** 1 Virus and Prion Disease Research Unit, National Animal Disease Center, USDA, Agricultural Research Service, 1920 Dayton Road, Ames, IA 50010, USA; 2 Agence Nationale de Sécurité Sanitaire (Anses)-Lyon, 31 Avenue Tony Garnier, 69364, Lyon Cedes 07, France; 3 Istituto Zooprofilattico Sperimentale Piemonte Liguria e Valle d'Aost, Via Bologna 148, 10154, Torino, Italy; 4 National & OIE Reference Laboratories for BSE NCAD/CFIA, Township Road 9-1 (Box 640), Lethbridge/Alberta, Canada

**Keywords:** Bovine spongiform encephalopathy, BSE, ELISA, Prion, PrP, Scrapie, Stability, Transmissible spongiform encephalopathy, TSE

## Abstract

**Background:**

Transmissible Spongiform Encephalopathies (TSEs), including scrapie in sheep, chronic wasting disease (CWD) in cervids, transmissible mink encephalopathy (TME), and bovine spongiform encephalopathy (BSE), are fatal diseases of the nervous system associated with accumulation of misfolded prion protein (PrP^Sc^). Different strains of TSEs exist, associated with different PrP^Sc^ conformations that can be probed by the stability assay, in which PrP^Sc^ is treated with increasing concentrations of the denaturant guanidine hydrochloride (GdnHCl).

**Results:**

Here, we provide the first comprehensive application of a rapid, protease-free version of the GdnHCl stability assay to brain tissue from cattle experimentally infected with various TSE isolates. Consistent with previous findings from a single Japanese isolate, the L-type isolates of BSE are not distinguishable from classical BSE in this assay. In contrast, H-type isolates of BSE, including our unique isolate of E211K BSE, exhibit higher stability than classical BSE, suggesting that its increased protection against protease digestion at the BSE N-terminus is associated with a higher stability in GdnHCl. While the difference in stability in our version of the assay is likely not large enough for effective use in a diagnostic laboratory setting, the use of alternative experimental conditions may enhance this effect. TSEs from other natural host species that have been passaged in cattle, including CWD and TME, were not distinguishable from classical BSE, while isolates of cattle passaged scrapie exhibited a slight increase in stability as compared to classical BSE.

**Conclusions:**

These results suggest that the core of PrP^Sc^, as probed in this assay, has similar stability properties among cattle-passaged TSE isolates and that the conformational differences that lead to changes in the proteinase K cleavage site do not cause large changes in the stability of PrP^Sc^ from TSE-affected cattle. However, the stability differences observed here will provide a basis of comparison for new isolates of atypical BSE observed in the future and in other geographic locations, especially in the case of H-type BSE.

## Background

Prion diseases, or transmissible spongiform encephalopathies (TSEs), are neurodegenerative, inevitably fatal diseases of mammals that include bovine spongiform encephalopathy (BSE), scrapie in sheep and goats, chronic wasting disease (CWD) in cervids, transmissible mink encephalopathy (TME), and Creutzfeldt-Jakob disease (CJD) in humans. The protein only hypothesis proposes that prion disease is associated with the misfolding of the normal cellular prion protein (PrP^C^) into a more protease-resistant, β-sheet rich conformation (PrP^Sc^) [1], which can be induced by infection with exogenous PrP^Sc^, spontaneous accumulation of PrP^Sc^, or genetic mutations in the prion gene (*PRNP*) [2].

Characterization of TSE isolates has provided evidence for distinct strains of particular prion diseases; for example, different strains of scrapie induce distinct pathologic profiles in rodent brain [3-5]. Strain differences are attributed to different conformations of the prion protein [6,7]. Conformational differences can be probed by western blot using proteinase K; some strains differ in the proteinase K cleavage point on the flexible N-terminus of PrP^Sc^, leading to differences in size and in their reactivity with monoclonal antibodies that recognize the N-terminus of PrP [8]. An alternative method of probing conformational differences involves denaturation of PrP^Sc^ in increasing concentrations of guanidine hydrochloride (GdnHCl) [9]. After incubation of PrP^Sc^ from infected tissues in GdnHCl, the fraction of PrP^Sc^ remaining is measured by methods such as proteinase K digestion coupled with western blotting or ELISA [9,10], the conformation-dependent immunoassay [11], or the conformational solubility and stability assay [12]. Much of the work characterizing strain stability has been completed on rodent-passaged TSE strains, but a growing number of studies are examining the biochemical properties of PrP^Sc^ obtained directly from livestock or human brain samples as a means of better understanding PrP^Sc^ in the natural host system [12-14].

While the UK BSE outbreak in cattle has been associated with oral transmission of the classical type of BSE (C-type BSE) *via* contaminated feed [15,16], increased global surveillance has identified two distinct forms of atypical BSE that are believed to arise spontaneously in older cattle: H-type BSE (named for a molecular weight shift 1–2 kDa higher for proteinase K-digested PrP^Sc^ on a western blot) and L-type BSE (named for a ≈ 0.5 kDa lower molecular weight) [17-20]. Type-specific differences in the ratio of PrP^Sc^ glycoforms present, the reaction pattern with different anti-PrP monoclonal antibodies, and the pattern of PrP^Sc^ accumulation in infected brains (as detected by immunohistochemistry) are also observed [21,22]. In addition, a case of H-type BSE was diagnosed in the U.S. that led to the identification of a new *PRNP* allele, K211 [23]. The E211K polymorphism is homologous to the E200K mutation, the leading cause of inherited CJD in humans, suggesting that this case is the first example of an hereditary TSE in livestock [24].

Here, we provide the first comprehensive application of the GdnHCl stability assay to cattle-passaged TSE isolates including classical (representing infectious prion disease in cattle), atypical (representing spontaneous prion disease in cattle), and E211K (potentially representing genetic prion disease in cattle) isolates of BSE, as well as cattle-passaged CWD, scrapie, TME, and bovine TME. In addition to the potential for these stability studies to aid in laboratory diagnostics, differences in stability could also suggest differences in inactivation potential between types of cattle TSEs and could speak to relationships between prion diseases.

## Methods

### Origin and processing of clinical samples

Samples of H-type, L-type, and classical BSE-infected obex (as well as obex from the 4^th^ passage of transmissible mink encephalopathy (TME) into cattle) were obtained from steers intracranially (IC) inoculated with 1 ml of 10% (w/v) brain homogenate (BH) of each of these isolates. The animal experiments were carried out in accordance with the Guide for the Care and Use of Laboratory Animals (Institute of Laboratory Animal Resources, National Academy of Sciences, Washington, DC) and the Guide for the Care and Use of Agricultural Animals in Research and Teaching (Federation of Animal Science Societies, Champaign, IL). The protocol was approved by the Institutional Animal Care and Use Committee at the National Animal Disease Center (protocol number: 3985). Animals were euthanized upon development of unequivocal clinical signs. Original sources of the inocula were as follows: French H and L-type BSE [25], U.S. classical and H-type BSE [26], Canadian L-type BSE [27], and Italian L-type BSE (BASE) [19]. We note that the numbers of biological replicates in this study are limited by the numbers of infected cattle brain samples available for analysis.

In a separate experiment, BSE-infected E211K brain homogenate (obtained from the 2006 U.S. BSE case [23]) was IC inoculated into a EK_211_ heterozygous calf (Animal #25); complete details of the experiment and the histopathology of calf #25 were described previously [28]. Experimental infection of a wild-type calf (Animal #26) with classical BSE brain homogenate was completed in parallel by IC inoculation.

Samples of cattle brains IC infected with mink TME (1^st^ passage of mink material into cattle), sheep scrapie, and CWD from white-tailed deer were obtained from previously published studies, in which the results of western blots and microscopic analysis are described elsewhere [29-31].

The incubation times of TSE disease in the animals used in this study are shown in Table 1. Brain homogenates from each animal were prepared as whole homogenates of brainstem sections by bead homogenization with 1.0 mm silica beads in 1X Dulbecco’s PBS pH 7.4 (lacking Ca^2+^ and Mg^2+^).

**Table 1:** Clinical Information for cattle experimentally infected with BSE and other TSEs

**Animal number**	**Inoculated TSE**	**Source of inoculum**	**Incubation time (days)**
**1**	Classical BSE	U.S. 2003 (WA)	532
**2**	Classical BSE	U.S. 2003 (WA)	715
**3**	Classical BSE	U.S. 2003 (WA)	639
**4**	Classical BSE	U.S. 2003 (WA)	631
**5**	H-type BSE	French case	469
**6**	H-type BSE	U.S. 2004 (TX)	505
**7**	H-type BSE	U.S. 2004 (TX)	504
**8**	L-type BSE	French case	475
**9**	L-type BSE	French case	462
**10**	L-type BSE	BASE (Italian)	554
**11**	L-type BSE	Canadian case	510
**12**	TME	4^th^ cattle passage	463
**13**	TME	4^th^ cattle passage	477
**14**	TME	4^th^ cattle passage	432
**15**	TME	4^th^ cattle passage	482
**16**	TME	Mink	430
**18**	TME	Mink	457
**19**	Scrapie	Sheep	537
**20**	Scrapie	Sheep	563
**21**	Scrapie	Sheep	624
**22**	CWD	White-tailed Deer	264
**23**	CWD	White-tailed Deer	252
**24**	CWD	White-tailed Deer	216
**25**^**a**^	E211K BSE	2006 U.S. BSE (AL)	301
**26**^**b**^	Classical BSE	U.S. 2003 (WA)	776

AL- Alabama, TX-Texas, WA- Washington.

^a^This calf had the EK_211_ genotype to match the genotype of the donor for inoculation.

^b^This calf had a wild-type genotype at position 211 (like the other animals in the table).

### Western blotting of proteinase-resistant BSE PrP^Sc^

Western blotting on the BSE samples was performed as described previously [14]. Briefly, brain homogenate prepared in 1X PBS (pH 7.4) was digested with 100 μg/ml of PK at 37°C for 1 hour, followed by preparation by boiling under denaturing and reducing conditions (1X LDS sample buffer (Life Technologies) + 5% β-mercaptoethanol). Samples were separated on a 12% Bis-Tris NuPAGE gel, electroblotted to PVDF, and probed with the 6H4 primary antibody for detection.

### IDEXX ELISA-based PrP^Sc^ stability curves

**Figure 1:**
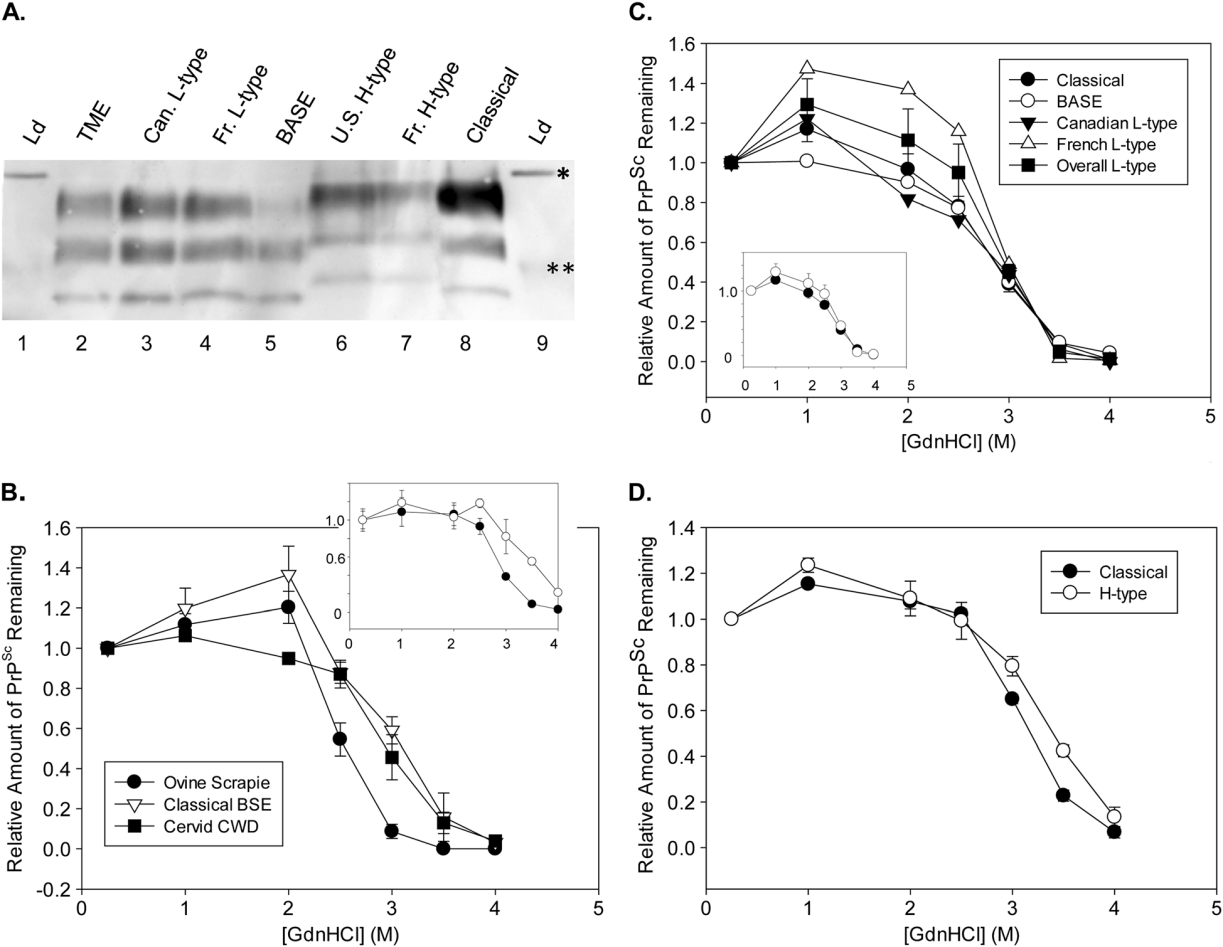
**Stability of BSE isolates. (A)**. Western blot of isolates passaged in cattle. Obex samples were characterized by PK- western blotting (mAb 6H4). Lanes from left: 1, Biotinylated protein marker; 2, Bovine TME (Animal #14, 0.5 mg); 3, Canadian L-type (#11, 0.5 mg); 4, French L-type (#8, 1 mg); 5, BASE (#10, 0.5 mg); 6, U.S. H-type (#7, 1 mg); 7, French H-type (#5, 1 mg); 8, Classical (#4, 1 mg); 9, Marker. Molecular weights of ladder bands were: *, 31 kDa; **, 21 kDa. **(B-D)**. Curves represent the average of biological replicates except where noted with 3 to 5 technical replicates per animal. The same stock of GdnHCl was used for curves that were directly compared. Error bars reflect standard error of the mean (SEM), except where indicated. **(B)**. Comparison of TSEs from natural hosts. Curves represent the average of 2-3 experiments from a representative animal; error bars represent standard deviation. Open triangles—Classical BSE (Animal #3); Closed circles—136-VDEP sheep scrapie [14]; Closed squares—U.S. elk CWD [34]. Inset: Comparison of U.S. scrapie isolates 136-VDEP (closed circles) and 13-7 (open circles). (Absolute [GdnHCl]1/2 values differ here due to differences in experimental conditions). **(C)**. Classical vs. L-type BSE. The L-type overall curve (closed squares) is averaged across 4 biological replicates, and the classical curve (closed circles) is the average of 3 biological replicates, using the standard method. The French L-type curve (open triangles) is the average of 2 samples and the BASE (open circles) and Canadian L-type (closed triangles) curves represent individual biological replicates. Inset: Classical (closed circles) and Overall L-type (open circles) curves are displayed separately for ease of viewing. **(D)**. Classical vs. H-type BSE. Curves represent averages of 3 biological replicates of each performed using the buffered method. Closed circles—Classical BSE; Open circles—H-type BSE.

**Figure 2:**
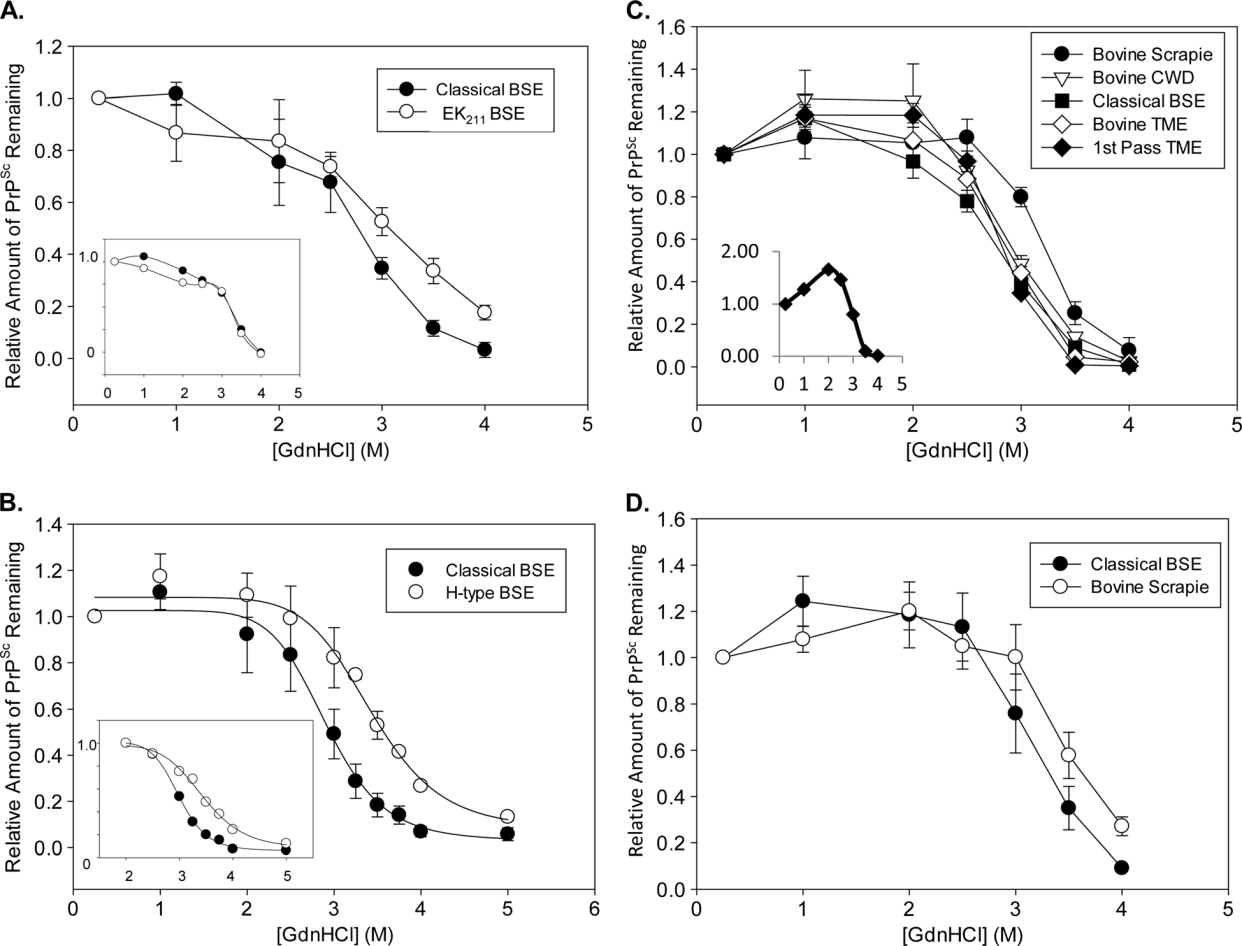
**Stability Properties of EK**_**211**_**, H-type, and Cattle-Passaged TSEs. (A)**. *Classical vs.* E211K *BSE Stability*. Closed circles—Classical BSE (Animal #26); Open circles—E211K BSE. Curves represent the average of 3 technical replicates for each animal, completed using the intermediate method; error bars represent standard deviation of replicates. The experiment was also completed independently using the buffered method, with the same stability pattern observed for the pair of samples (data not shown). ***Inset***: Comparison of stability curves of obex PrP^Sc^ (closed circles) and cerebellar PrP^Sc^ (open circles) from the EK_211_ animal (#25). **(B)**. *Detailed comparison of classical and H-type stability.* Data was collected with the intermediate method and an increased number of data points were taken between 0.25 M and 5 M GdnHCl. Error bars represent SEM and the H-type average curve includes the EK_211_ H-type sample. ***Inset:*** As described in the text, the curves in the main panel were normalized to the value at 2 M GdnHCl to display data between 2 and 5 M GdnHCl; closed circles—classical BSE average; open circles—H-type BSE average. Curves for both the main panel and the inset were fit with a 4-parameter logistic function (SigmaPlot). **(C)**. *Stability comparison of other TSEs passaged in cattle brain to classical BSE.* All curves represent the average of 3 biological replicates (error bars represent SEM), with the exception of the TME (1^st^ passage in cattle) curve, which displays the average of 2 biological replicates (error bars represent range). Curves were performed with the standard method. ***Inset***: Example of single TME isolate with significantly distorted stability profile, as described in Results. **(D)**. *Comparison of classical BSE and bovine scrapie.* Curves represent the average of 3 biological replicates (error bars represent SEM), performed using the intermediate method.

A detailed methodology for the determination of PrP^Sc^ stability by the use of the IDEXX HerdChek BSE ELISA (in the absence of proteinase K digestion) is provided in [14]. This method was modified as described below in order to address specific experimental requirements. In the standard method, 1X PBS-homogenized brainstem (at a final concentration of 2% or less) was incubated with 0.25-4 M GdnHCl in a 10 μl volume for 1 hr at room temperature, followed by dilution of all samples to 0.25 M GdnHCl in 1X PBS and detection of the level of PrP^Sc^ remaining by ELISA. The bovine anti-PrP conjugate was used for the ELISA protocol in all cases except for the scrapie in sheep brain and the CWD in cervid brain samples (Figure 1B), in which case the small ruminant anti-PrP conjugate was used for detection. 8 M GdnHCl solution was prepared by adding a 1X PBS pH 7.4 solution to GdnHCl powder, and the pH was adjusted to approximately pH 7 by the addition of NaOH. Curves that are directly compared in the graphs displayed here were completed with the same GdnHCl stock to control for small differences in GdnHCl stock concentration and final stock solution pH. [GdnHCl]_1/2_ values were defined as the concentration of GdnHCl required to reduce the level of the PrP^Sc^-specific signal by half (as compared to the signal at the 0.25 M GdnHCl data point). Due to variations in the shape of the upper baseline of curves, [GdnHCl]_1/2_ values in Table 2 were determined by the use of the Smooth Line function in Microsoft Excel to connect data points and visualize the [GdnHCl]_1/2_ value. In Figure 2B, additional curve fitting was performed using the 4-parameter logistic fit in SigmaPlot (Systat Software, San Jose, CA) as described in more detail in Results and Discussion.

**Table 2:** **Comparison of [GdnHCl]**_**1/2 **_**values for isolates under each experimental condition**

**Experimental conditions**	**TSE isolate in cattle**	**Average [GdnHCl]**_**1/2**_
**Standard**	Classical BSE	2.9 ± 0.04 M
**Standard**	L-type BSE	2.9 ± 0.05 M
**Standard**	TME (Cattle-passaged)	2.9 ± 0.03 M
**Standard**	CWD	3.0 ± 0.04 M
**Standard**	Scrapie	3.3 ± 0.04 M
**Buffered**	Classical BSE	3.2 ± 0.02 M
**Buffered**	H-type BSE	3.4 ± 0.03 M
**Intermediate**	Classical BSE	2.9 ± 0.1 M
**Intermediate**	H-type BSE (including E211K isolate)	3.5 ± 0.1 M

[GdnHCl]_1/2_ values are expressed as the average of data from 3 or more independent animals infected with each TSE type ± the standard error of the mean, with the exception of the E211K BSE case, for which only one animal sample is in existence worldwide. Experimental conditions refer to the buffer system used and are described in detail in the Methods. The intermediate method [GdnHCl]_1/2_ values described here do not include the classical vs. scrapie in cattle comparison conducted separately and displayed in Figure 2D. However, we note that if the biological and technical replicates used in that experiment are aggregated with the classical BSE numbers displayed here, the values for classical BSE and scrapie in cattle brain would be 3.0 ± 0.1 and 3.6 ± 0.1, respectively. The H-type BSE [GdnHCl]_1/2_ value would remain significantly different from the classical BSE [GdnHCl]_1/2_ at p < 0.05, and the scrapie in cattle [GdnHCl]_1/2_ value would then be significantly different from the classical BSE [GdnHCl]_1/2_ at p < 0.05.

Previous studies have demonstrated that in some cases 1X PBS is unable to fully buffer the lower pH found in prion-infected brain homogenate (BH) [32]; therefore, adjustments were made in the procedure (buffered method; Figure 1D) in cases where we needed to complete stability curves at high concentrations of BH. A final BH concentration of up to 10% was used; brainstem samples that had higher concentrations of PrP^Sc^ (and therefore required pre-dilution) were diluted into uninfected cattle BH. BH as well as the 8 M GdnHCl stock in this case were prepared in modified PBS (final pH = 7.9-8.0) containing 89 mM Na_2_HPO_4_, 15 mM KH_2_PO_4_, and 136.9 mM NaCl (for increased buffering capacity without increasing the NaCl concentration); the pH of the 8 M GdnHCl stock was adjusted with the addition of NaOH. (Since salt and pH conditions therefore differ between experimental conditions, GdnHCl curves cannot necessarily be compared across panels). For Figures 2A, B, and D, an intermediate experimental condition (intermediate method) was used in which brain homogenates were prepared in modified PBS (to increase buffering capacity), but the final concentration of BH in the reaction was ≤ 2%, and the rest of the reaction was prepared in 1X PBS as described in the standard method.

## Results and discussion

Brain samples were obtained from cattle experimentally inoculated (IC) with various isolates of classical and atypical BSE. Animals were euthanized upon development of clinical signs (see Table 1 for a comparison of incubation times). To confirm that strain-specific properties of each inoculum were transmitted to the experimental animals, obex tissue from selected animals was PK-digested, and PrP^Sc^ was detected on a western blot with the 6H4 antibody (Figure 1A). H-type and L-type samples are positively identified by differences in electrophoretic mobility on the blot as compared to classical BSE, and the ratios of the glycosylated forms of PrP^Sc^ for each sample are also consistent with the expected patterns for each strain. The appearance of PrP^Sc^ from the experimentally-infected animals was consistent with that of inoculated material (data not shown).

In order to rapidly and directly measure strain stability in BSE-infected brain, we utilized a method in which the fraction of PrP^Sc^ remaining at increasing GdnHCl concentration is measured with a commercial ELISA that specifically recognizes PrP^Sc^ (but not PrP^C^ or denatured PrP^Sc^). This methodology has previously been used to distinguish between different isolates of scrapie in sheep brainstem [14], but until now has not yet been applied to cattle. Here, cattle brainstem homogenate was incubated with 0.25-4 M GdnHCl, and the proportion of PrP^Sc^ remaining at each concentration was detected by the ELISA after dilution of the samples to low final GdnHCl concentration.

### Comparison of classical, H-type, and L-type BSE PrP^Sc^ by the stability assay

Initially, classical (or C-type) BSE was compared with other TSEs in their natural animal hosts by our standard stability assay. The stability curve for classical BSE samples was found to be approximately sigmoidal, consistent with curves obtained for other TSEs (Figure 1B) [12,14,33]. The stability of an elk CWD isolate [34] was similar to the stability of classical BSE, whereas a United States isolate of sheep scrapie (136-VDEP; [14]) had a lower stability, demonstrating the ability of this methodology to distinguish between isolates with different stability profiles (Figure 1B). Similarly, the stability of the 136-VDEP isolate (Figure 1B inset, closed circles) is distinct from the stability of a different U.S. scrapie isolate, 13–7 (Figure 1B inset, open circles), in this assay [14,35]. These results are consistent with previously reported results in mice; previous studies conducted by others determined that the [GdnHCl]_1/2_ values of (protease-resistant) PrP^Sc^ from transgenic mice inoculated with BSE or sheep scrapie were 2.8 and 2.2 M, respectively [33]. Results for scrapie might be expected to vary depending on the specific isolate inoculated.

As compared to classical BSE PrP^Sc^, PrP^Sc^ from cattle infected with L-type BSE from French and Italian (BASE) sources exhibited similar behavior in the steep region of the stability curve; however, some samples (derived from the French L-type inoculated cattle) exhibited a striking increase in ELISA signal at low GdnHCl concentrations (Figure 1C). While the specific cause of the increased ELISA signal at low GdnHCl concentration is not understood, the simplest explanation for this observation is either increased binding to the capture surface and/or enhanced exposure of the epitope for the detection antibody. With the exception of the effects noted in the upper baseline of the curve, the stability curve profile of classical BSE (Figure 1C, closed circles) was indistinguishable from that of L-type BSE (averaged over all L-type isolates; Figure 1C, closed squares). Due to the curve shape and the deviation from a perfect logistic fit, [GdnHCl]_1/2_ values (or the [GdnHCl] required to reduce the level of the PrP^Sc^-specific signal by half) were determined for isolates based on a smooth curve fit (Table 2). [GdnHCl]_1/2_ values for classical (2.9 ± 0.04 M) and L-type (2.9 ± 0.05 M) were also indistinguishable. This result is in agreement with that from a single isolate of Japanese L-type BSE in a different, proteinase K-dependent assay; when the stability of Japanese L-type BSE was compared to classical BSE, [GdnHCl]_1/2_ values were not significantly different (3.1 ± 0.1 M and 2.9 ± 0.3 M, respectively) [36].

Since many of the samples of H-type brainstem, as well as of H-type colliculus, from our experimental inoculation exhibited lower absorbances in the ELISA-based assay, the final concentration of BH in the assay was increased to 10% for a comparison with classical BSE. Some modifications in assay conditions (Buffered Method) were used to control potential variables across the experiment (including effects of brain pH and other brain components on the reaction). Using this methodology, an increase in the stability of H-type BSE (Figure 1D, open circles) was observed as compared to the stability of classical BSE (Figure 1D, closed circles). While this observed increase was small, the average [GdnHCl]_1/2_ value for three independent classical BSE samples (3.18 ± 0.02) was significantly different than the average [GdnHCl]_1/2_ of 3.40 ± 0.03 for the three independent H-type BSE samples (student’s unpaired t-test, p = 0.003; Table 2). Therefore, the increased size of the PK-resistant core in H-type PrP^Sc^ is associated with a somewhat higher stability as determined by GdnHCl unfolding. We note that this is merely an association, and the increased size is not necessarily causative with regard to the increased stability.

### Stability properties of E211K PrP^Sc^

Next, we examined the properties of BSE PrP^Sc^ obtained from passage of an H-type isolate of BSE from a cow with a polymorphism at codon 211 of *PRNP* (U.S. 2006 BSE case) to a calf also heterozygous for the E211K polymorphism [19]. (As this PrP^Sc^ may contain a mixture of E211 and K211 protein, we refer to this isolate as E211K and cattle carrying the mutation as EK211). This isolate serves as a potential example of a genetic TSE in a non-human species, as well as an additional example of an H-type BSE transmission. PrP^Sc^ material from this passage has a western blot profile consistent with that of H-type BSE [28]. Using our standard assay conditions, a higher stability of the passaged E211K PrP^Sc^ than for classical PrP^Sc^ from the brain of a wild-type calf was observed (data not shown). A test of 20% classical BH (Animal #3) and EK_211_ BH (Animal #25) on pH paper indicated that the pH of each was slightly lower than the pH of our 1X PBS (originally pH 7.4); while this suggests that acidification was present in the brain tissue, we note that no differences in pH between the homogenates were evident in this crude assay.

To further confirm this result while controlling for the potential pH variable, EK_211_ brain sample (Animal #25) was homogenized in a version of PBS buffer with 10X buffering capacity and compared to a PrP wild-type calf that had been inoculated with classical BSE (Animal #26) in parallel; an increase in stability of the EK_211_ PrP^Sc^ was observed in this version of the assay as well (Figure 2A). We note that we are limited in our ability to assess the statistical significance of this result on its own, as only one biological sample of passaged E211K is available worldwide. However, this is the first assessment of the PrP^Sc^ stability of a purported hereditary TSE in a non-human species.

Previous Proteinase K-western blot analysis of regions of the EK_211_ (Animal #25) brain identified a difference between the pattern of PrP^Sc^ from the obex and from the cerebellum, with an additional 23 kDa band apparent in the EK_211_ cerebellum only (which is not observed in other H-type samples) [28]. We compared EK_211_ obex (Fig. and cerebellum PrP^Sc^ in the stability assay, but did not observe a difference between these tissues (Figure 2A, inset).

### Additional evidence for the increased stability of H-type BSE PrP^Sc^

Since the pattern of increased PrP^Sc^ stability for H-type BSE samples was similarly observed in our H-type E211K isolate, we wanted to provide additional evidence to support our conclusion that this higher stability was statistically significant. Therefore, we utilized a slightly different experimental condition (Intermediate Method) paired with the use of more data points (from 0.25-5 M GdnHCl) with the aims of better establishing the difference between the [GdnHCl]_1/2_ values and better defining the stability curve for our samples. Since this method necessarily involved the use of a lower final concentration of BH, we identified two H-type samples with strong enough signal in the ELISA for analysis (Animals #6 and #7, representing U.S. H-type BSE) and compared their stability curves to those of three classical BSE isolates. We combined this data with data collected from animal #25 (E211K BSE) as a third example of H-type BSE tested under the intermediate experimental conditions, as well as with animal #26 (as another example of classical BSE) to generate the curves in Figure 2B. Using our previous methodology (Table 2), the average [GdnHCl]_1/2_ value from the classical BSE group (2.9 ± 0.1 M) was significantly different from the average [GdnHCl]_1/2_ value from the H-type BSE group (3.5 ± 0.1 M) (student’s unpaired t-test, p = 0.02).

Since a logistic fit has been used to analyze GdnHCl stability curves in previous studies (i.e. [9,12]), the average H-type curve (Figure 2B, open circles) and the average classical type curve (Figure 2B, closed circles) are also displayed here with 4-parameter logistic fits to further demonstrate the difference between the isolate types. Finally, since much of the deviation from a logistic fit occurs between 0.25 and 2 M Gdn for our samples, we also normalized these curves to the ELISA value at 2 M GdnHCl and performed logistic fits (Figure 2B, inset; average H-type curve in open circles and average classical curve in closed circles). When we performed the same normalization on the curves from the independent biological replicates, the calculated [GdnHCl]_1/2_ values from these fits were 3.49 and 3.42 for the H-type BSE isolates and 3.06, 2.96, and 2.95 for the classical BSE isolates. In combination with the data and analysis of Figure 1D, we believe that this provides additional confirmation of our reported higher stability of H-type BSE.

### Stability comparison of BSE to other cattle-passaged TSEs

In addition to comparing the different BSE strains, we also used the stability assay to characterize the biochemical properties of other TSEs passaged into cattle. Scrapie and CWD are both transmissible into cattle by IC inoculation, leading to PrP^Sc^ accumulation--but not significant spongiform changes--in the brain [29,30]. Transmissible mink encephalopathy has been hypothesized to have originated from the feeding of downer cattle, possibly carrying atypical, L-type BSE, to farm-raised mink [37]. We wanted to determine if the profiles of PrP^Sc^ from these TSEs passaged in cattle brain were distinguishable from each other or from other BSE strains, with potential implications for understanding strain origins and/or improving (non-BSE) TSE diagnosis in cattle.

Stability curves for white-tailed deer-derived CWD on the first passage into cattle (Figure 2C, open triangles) matched the profile of classical BSE PrP^Sc^ (Figure 2C, closed squares), which is not surprising due to the similarity of the stability of classical BSE and CWD in their natural hosts (Figure 1B). For TME, samples from a 4^th^ passage of a TME isolate into cattle (Bovine TME; performed in parallel to the BSE strain inoculation experiment) were analyzed. Of the four biological replicates analyzed, one sample exhibited a significant deviation from the baseline (Figure 2C, inset). Possibly, this reflects differences in higher-order PrP^Sc^ structure, or another biochemical difference in this sample. The other three TME samples were averaged to create the stability curve in Figure 2C (open diamonds), which matches the average curve for classical BSE (Figure 2C, closed squares). [GdnHCl]_1/2_ values for CWD in cattle (3.0 ± 0.04) and for Bovine TME in cattle (2.9 ± 0.03) were indistinguishable from that of classical BSE. To determine if the stability of TME had changed upon repeated passage into cattle, we compared this 4^th^ passage stability curve to that of cattle infected directly with mink TME. The 1^st^ passage of TME from mink into cattle generated PrP^Sc^ with a stability profile consistent with both 4^th^ passage TME and classical BSE (Figure 2C). We note that the previous studies of passage of TME into cattle did not note a decrease in disease incubation time between the original and second passages into cattle, suggesting little or no species barrier between the mink TME and the cattle prion protein [31].

In the case of sheep scrapie inoculated into cattle, the average curves over three available animals (Figure 2C, closed circles) showed a small increase in stability ([GdnHCl]_1/2_ = 3.3 ± 0.03 M) as compared to the stability of classical BSE (Figure 2C, closed squares; [GdnHCl]_1/2_ = 2.9 ± 0.04). The [GdnHCl]_1/2_ values from this experiment were significantly different using the unpaired student’s t-test at p < 0.05. However, due to the comparatively low absolute ELISA values for two of the bovine scrapie samples, we elected to conduct additional analysis using the intermediate method (see Methods) to better compare the stability of bovine scrapie to that of classical BSE with curves conducted in parallel on the same plate. Again, a small increase in stability was observed for the scrapie isolate in cattle brain (Figure 2D, open circles; [GdnHCl]_1/2_ = 3.6 ± 0.2) as compared to the stability of classical BSE in cattle brain (Figure 2D, closed circles; [GdnHCl]_1/2_ = 3.2 ± 0.1). However, a higher than average standard error of the mean was observed in this experiment for the classical BSE data set, and the difference in stability was significant at the p = 0.10 but not the p = 0.05 level. One major challenge of the bovine scrapie analysis was the issue with low signals in some of the other available samples attempted, limiting our sample population for analysis. Therefore, we suggest that it is likely that the bovine scrapie PrP^Sc^ has a slightly higher stability than classical BSE, and that further analysis of these isolates upon future passage would be instructive for continued investigation of these relationships.

Previous work by others demonstrated that the stability of BSE PrP^Sc^ ([GdnHCl]_1/2_ = 2.78 M) when passaged into transgenic mice expressing bovine PrP was significantly higher than the stability of scrapie PrP^Sc^ passaged into these mice ([GdnHCl]_1/2_ = 2.25 M) [38] even though the stability of PrP^Sc^ from the original scrapie-infected sheep brain material was similar to the stability of PrP^Sc^ from the BSE-infected cattle brain material (resistant to > 3.0 M GdnHCl). The original conclusion was that bovine scrapie PrP^Sc^ has a lower stability than bovine BSE PrP^Sc^; however, our results lead us to propose that different isolates of scrapie induce different stability profiles following passage in cattle. Alternatively, this result could be due to differences in the molecular properties probed by the two different versions of the GdnHCl stability assay. We have observed changes in the stability profile of some scrapie isolates when compared in the presence and absence of proteinase K digestion (C.E. Vrentas, unpublished), and the same phenomenon has been observed in human CJD strains [39]. The original scrapie inoculum for the cattle in this study, which consisted of a pool of nine U.S. field cases from 1992, was not available for testing. However, as the stability of the U.S. 13–7 scrapie isolate in sheep brain was similar to the stability of BSE but not of the U.S. 136-VDEP isolate (compared under parallel conditions, data not shown), we propose that the scrapie isolate(s) inoculated into to the infected cattle used here could have similar properties to the 13–7 strain.

## Conclusions

Overall, this study provides the first comprehensive comparison of the GdnHCl stability properties of TSE PrP^Sc^ as passaged into cattle, utilizing a version of the assay that probes PrP^Sc^ present in the whole, unfractionated homogenate. In addition to examining multiple, geographically distinct isolates, our assay provides a more complete picture of the PrP^Sc^ present in the bovine brain tissue, including both PK-sensitive and PK-resistant fractions. This is potentially important for the comparison of classical and atypical BSE, as atypical BSE PrP^Sc^ samples exhibit increased sensitivity to PK [22,27]. While the characterization of cattle brain samples directly is challenging due to the limitations in sample numbers, the use of this rapid, easy-to-quantify assay combined with our unique collection of cattle TSE isolates has allowed for an increased understanding of PrP^Sc^ properties in the natural host.

In our comparisons of BSE isolates, we determine that the increased protection against PK digestion at the N-terminus of H-type BSE PrP^Sc^ correlates with a higher stability in GdnHCl as compared to classical BSE. Additionally, we provide the first stability curves for what is believed to be an hereditary form of BSE associated with the K211 allele of *PRNP*, and demonstrate that the stability of PrP^Sc^ from the EK_211_ animal is consistent with its H-type profile in the western blot. From a diagnostic standpoint, however, this difference in stability does not appear to be large enough for a rapid test on an ELISA plate (such as a +/- GdnHCl pre-treatment test).

In contrast, L-type BSE stability profiles did not differ relative to classical BSE as determined here. Changes in signal at low GdnHCl concentration appeared to be type and possibly sample-specific and may be of interest for future investigations into the structural reasons for these differences between PrP^Sc^ aggregates. Our [GdnHCl]_1/2_ results for L-type vs. classical BSE are consistent with previous results for a single Japanese isolate of L-type BSE [36]. We suggest that this version of the stability assay can serve as an interesting means of characterizing future cases of atypical BSE around the world, in order to determine if the H-type BSE characterization by other methods is always associated with a higher GdnHCl stability profile, or if there is any diversity in stability profile observed for other classical and L-type BSE isolates. The method will also serve as a means to track PrP^Sc^ properties, alongside western blotting, upon continued serial passage of these isolates in cattle.

Results from stability studies with TME, bovine passaged TME, and white-tailed deer CWD indicate that these diseases in cattle would, like L-type BSE, be indistinguishable from classical BSE in this assay. In the case of sheep scrapie in cattle, we observe a stability profile for PrP^Sc^ that is at least as stable as (and likely more stable than) PrP^Sc^ from cattle with classical BSE, a result distinct from those obtained in studies using scrapie and BSE passaged in bovinized transgenic mice [38]. We propose that different isolates of scrapie lead to different PrP^Sc^ conformations and associated stabilities upon passage to cattle; therefore, the stability assay would not necessarily be able to distinguish bovine scrapie and BSE in the diagnostic laboratory. Further stability investigation of bovine-passaged scrapie in cattle and mouse systems, as well as in *in vitro* conversion systems, is warranted in order to understand the implications of scrapie isolate or strain diversity on resultant properties in the converted cattle protein. The presence of two distinct disease phenotypes has been demonstrated in cattle IC inoculated with scrapie isolates from Great Britain [40]. As both groups of cattle exhibited significant vacuolar change, these phenotypes may be distinct from that of the U.S. scrapie in cattle [29]. Only one of the Great Britain cattle scrapie phenotypes was associated with a higher molecular weight than classical BSE as observed by proteinase K-Western [40], and we suggest that it would be instructive to compare the stability profiles of each of these bovine passaged scrapie.

Finally, we note that the use of detergent treatment as an alternative means of examining the biochemical properties of BSE in cattle reveals a different pattern between classical and atypical BSEs. Namely, the use of detergent to probe the structural stability of PrP^Sc^ revealed that classical BSE was more resistant to detergent treatment (as probed over a range of detergent types) than was German L-type BSE [41]. As noted by Breyer et al. [41], the detergent treatment affects protein stability through hydrophilic and hydrophobic interactions, as compared to the effect of GdnHCl which may influence hydrogen bonding. Proteinase K treatment was used subsequent to detergent treatment, similar to the use of PK after GdnHCl treatment in some other forms of the stability assay. The detergent finding is perhaps consistent with the fact that both H-type and L-type BSE are significantly more sensitive to PK digestion under stringent reaction conditions than classical BSE [22]. However, the proteinase K sensitivity of a PrP^Sc^ isolate is not necessarily correlated with the stability of that isolate to GdnHCl denaturation. We previously observed similar PK sensitivity profiles but significantly different GdnHCl stability profiles in the whole homogenate assay in the case of U.S. sheep scrapie isolates [14]. Also, the GdnHCl stability profile under our assay conditions is a combination of the profiles of PK-resistant and more PK-sensitive fractions. At this time, it is not understood exactly what the GdnHCl stability curve is probing: loss of tertiary structure, disruption of the quaternary structure of aggregates, or both. We suggest that further comparison of the results of these different assays will provide us with more information about the differences in prion structure and aggregation patterns that cause the strain-dependent differences. The effect of the lipid component of PrP^Sc^ aggregates [41] is of particular interest, and further studies with the rapid stability assay will consider the impact of detergent treatment and lipid extraction on stability of isolates in GdnHCl.

## Abbreviations

BASE: Bovine amyloidotic spongiform encephalopathy; BSE: Bovine spongiform encephalopathy; CWD: Chronic wasting disease; IC: Intracranial; GdnHCl: Guanidine hydrochloride; PRNP: Prion protein gene; TSE: Transmissible spongiform encephalopathy; TME: Transmissible mink encephalopathy.

## Competing interests

The authors declare that they have no competing interests.

## Authors’ contributions

CEV designed the study, performed experiments, and prepared the manuscript; JJG contributed animal specimens for analysis, provided clinical information and interpretation, and helped draft the manuscript; TB, MC, and SC contributed essential reagents; EMN conceived the experiment, helped interpret molecular data and draft the manuscript. All authors read and approved the final manuscript.
